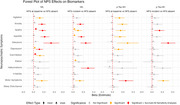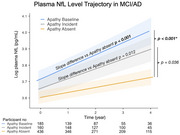# Apathy and Affective Symptoms are Associated with Elevated Alzheimer's Disease Biomarkers

**DOI:** 10.1002/alz70856_102721

**Published:** 2025-12-24

**Authors:** Matthew JY Kang, Dhamidhu Eratne, Christa Dang, Dennis Velakoulis

**Affiliations:** ^1^ University of Melbourne, Parkville, VIC, Australia; ^2^ Neuropsychiatry Centre, The Royal Melbourne Hospital, Parkville, VIC, Australia; ^3^ National Ageing Research Institute, Melbourne, VIC, Australia

## Abstract

**Background:**

Apathy and affective neuropsychiatric symptoms (NPS) are prevalent in Alzheimer's disease (AD), yet their neurobiological correlates are not fully understood. We examined associations between plasma markers of neurodegeneration (neurofilament light chain, NfL) and tau pathology (*p*‐tau181) with apathy and affective symptoms in mild cognitive impairment (MCI) and AD dementia.

**Method:**

This longitudinal study analyzed data from 781 participants with MCI and AD dementia enrolled in the Alzheimer's Disease Neuroimaging Initiative (ADNI), with annual blood samples collected over four years. Neuropsychiatric symptoms were assessed via the Neuropsychiatric Interview (NPI), and biomarker trajectories were analyzed using mixed‐effects models.

**Result:**

Elevated plasma *p*‐tau181 and NfL levels were associated with apathy and anxiety in MCI and AD dementia, with apathy linked to a significantly higher rate of NfL increase, indicating accelerated neurodegeneration.

**Conclusion:**

Apathy and anxiety may indicate greater tau and neurodegenerative burden in AD, highlighting potential targets for treatment.